# The Poor Man's Home.—XI

**Published:** 1897-10-30

**Authors:** 


### Modern Sociology.

### THE POOR MAN'S HOME.—XI.

### Master and Man.

The housing of workers by their employers is not a new thing. It is rather a return to the old idea of the master's obligations. When the worker was the slave it was part of his master's duty to house as well as to feed and clothe him. If this housing was bad, it was only because in the days of house-slavery the notion of healthy living was in its infancy, and, from a sanitary point of view, the lord's home was often little better than the serf's. Even then it is probable that the poor freedman was worse off in every way than the serf. The fact that men sold their children and even themselves into slavery shows that the life of the slave was not then regarded as one of abject misery. In this, as in many other things, it is probable that the abolition of slavery did little for the material welfare of the working class. But even when the formal responsibility involved in the relation of master and slave was lost, there still remained a tacit notion that the master should see that his worker had a roof over his head. In the primitive industry, farming, it is still a matter of course that the ploughman, the shepherd, and most of the other workers should have houses provided for them. It is reckoned as part of their wage, and, as farmers know, men will change their situation as often because of a superiority in the house as because of a larger wage.

The accommodation thus provided often leaves a great deal to be desired. In the country ideas of sanitation progress less quickly than in towns, perhaps because the abundance of fresh air makes careful sanitation of less moment than in more crowded districts. And it must be admitted that the case of the married farm labourer, although not enviable, is better than that of the worker in many other industries. His house, if kept in proper repair, is not, as a rule, unhealthy; he has a garden, he has a healthy environment. Damp is the one great foe in these houses; and, as agriculture stands at present, there is more slackness than one would fain see in the building of houses on a solid foundation, and with their first floor raised sufficiently above the level of the soil. Earthen floors are now almost unknown, but in many places you still step down, not up, from the road into the house. This, as well as the fact of outdoor labour in all weathers, must be taken into account in explaining the prevalence of rheumatism among the rural labouring class. The provision for unmarried workers on farms is, however, often very unsatisfactory, especially those casual workers who are employed only at haymaking or harvest, or such busy seasons. Their demands in this matter are certainly not excessive, and if they have a roof to sleep under at all, they are not in haste to complain. But the huddling together in lofts and barns has often led to epidemics, and is always objectionable on the score of morality.

In industries which are carried on in districts remote from the crowded centres, the responsibility of providing housing still rests with the employer. At present the compulsion to provide houses for their workers affects only those who deal with natural products—the owners of coal-pits, quarries, and mines of every sort. It must be owned that the provision made by many of these is unsatisfactory. These industrial villages are wholly lacking in beauty of any kind. The houses are run up cheaply, with but little regard for either convenience, health, or decency. In the beginning of an enterprise it is comprehensible that a capitalist may be unwilling to add to the expense by spending more than he can help on houses which may not be long wanted; but this unfortunately makes bad housing almost a matter of course, and long after the venture has turned out prosperously, the workers are living in crowded and insanitary homes. The pressure of public opinion, however, is working in this matter. Employers are themselves growing to perceive that a good home tends to make a man healthy, therefore sober, therefore reliable.

The most famous industrial town of the new and wholesome kind is, perhaps, Pullman, near Chicago, built by the inventor of the Pullman railway car. In towns thus built *ab initio* with one object, all classes share in the advantages of good planning, good draining, and the like, which are more easily, and proportionately more cheaply, arranged for on a large scale. Mr. Pullman has built both tenements and separate houses. A tenement block affords accommodation for 12 families. There are eight flats of three rooms each. In these the living room measures 15 ft. by 13 ft.; one bedroom is 15 ft. by 7 ft. 6 in., and the other 12 ft. in length, and as wide as the first. The block contains also four flats of four rooms each. In these the kitchens measure the same as the living-room in the smaller flats, and there is in addition a parlour measuring 15 ft. by 7 ft. 6 in. One bedroom is 12 ft. long and 8 ft. wide, and the other measures 9 ft. by 9 ft. 6 in. The ceilings are from 8 ft. 6 in. to 9 ft. in height. This seems rather low-roofed; and we cannot think that the bedrooms in the four-room houses should be so small. In the endeavour to divide the same floor space into four rooms as is occupied by the three room flats, comfort has been sacrificed to the vanity of having two sitting-rooms. From an English point of view the rents are high. A three-room flat costs £1 12s. 6d. per month, and one of four rooms £1 16s. It is estimated that the rent amounts to about 20 per cent. of the earnings of the head of the family. Even allowing for food being cheaper in America than here, one cannot but feel that, considering the high price of clothing and manufactured articles of all sorts, this rental must be a serious item in the working man's budget. We are assured, however, that these tenements are very popular, and are always occupied.

The separate houses are of various kinds, their rentals running from £3 2s. 6d. to £10 per month. The latter of these hardly come under our observation; the man who can pay £120 a year in house rent cannot by any stretch be reckoned among the poor.

One of the typical smaller houses contains five rooms. It has a frontage of 17 ft., and a depth of 32 ft. The living rooms are large, a kitchen measuring 16 ft. by 14, and a parlour which is 16 by 12. One bedroom also is of fair size, 16 ft. by 12; but the two others are very small—only 7 ft. by 8 ft. But the American has not the Englishman's love of fresh air, and can be comfortable where one of us would be stifled.

